# Motion-induced compression of perceived numerosity

**DOI:** 10.1038/s41598-018-25244-8

**Published:** 2018-05-03

**Authors:** Michele Fornaciai, Irene Togoli, Roberto Arrighi

**Affiliations:** 1 Department of Psychological & Brain Sciences, University of Massachusetts, Amherst, MA USA; 2 Department of Neuroscience, Psychology, Pharmacology and Child health (NEUROFARBA), University of Florence, via di San Salvi 12, Firenze, 50139 Italy

## Abstract

It has been recently proposed that space, time, and number might share a common representation in the brain. Evidence supporting this idea comes from adaptation studies demonstrating that prolonged exposure to a given stimulus feature distorts the perception of different characteristics. For example, visual motion adaptation affects both perceived position and duration of subsequent stimuli presented in the adapted location. Here, we tested whether motion adaptation also affects perceived numerosity, by testing the effect of adaptation to translating or rotating stimuli moving either at high (20 Hz) or low (5 Hz) speed. Adaptation to fast translational motion yielded a robust reduction in the apparent numerosity of the adapted stimulus (~25%) while adaptation to slow translational or circular motion (either 20 Hz or 5 Hz) yielded a weaker but still significant compression. Control experiments suggested that none of these results could be accounted for in terms of stimulus masking. Taken together, our results are consistent with the extant literature supporting the idea of a generalized magnitude system underlying the representation of numerosity, space and time via common metrics. However, as changes in perceived numerosity co-varied with both adapting motion profile and speed, our evidence also suggests complex and asymmetric interactions between different magnitude representations.

## Introduction

The environment we live in continuously supplies us with an extraordinary amount of sensory information. Amongst all, cues about the position, time of occurrence and numerosity of the stimuli play a fundamental role in guiding our behavior. Indeed, they define *where* the objects are located, *when* the events occur and *how many* stimuli we have to deal with. Not only do spatial, temporal and quantity information have to be processed in parallel at the same time but, in most circumstances, this information has to be merged together to let us interact efficiently with the events in the environment. In line with this, it has been proposed the existence of common perceptual mechanisms, allegedly to be located in partially overlapping brain regions, which would encode space, time and number with similar metrics. For example, according to the Theory of magnitude (ATOM), different quantities would be processed by means of a common machinery called Generalized Magnitude System^1,2^.

This idea of a common magnitude system is supported by much psychophysical, physiological and imaging evidence (for reviews see:^1,3^) revealing interference between the estimation of a given stimulus property and the magnitude of different unrelated features. For instance, interactions amongst processing of spatial and temporal information are revealed by large stimuli being perceived to last longer than their actual duration and vice versa^4–8^. Similarly, interferences between estimates about stimulus duration and its numerosity have been reported when numerosity was defined by the number of impulses in a sequence^9^ as well as by an abstract representation via Arabic numerals^7^. Even more striking are the interactions between space and numbers. As highlighted by Sir Frances Galton in his 1880 publication, numbers seem to be represented in the brain along a line: the mental number line^10^. Despite spatial mapping of numbers robustly differs across individuals (with reports ranging from a straight line to a completely irregular space), the idea of an intimate link between space and number has been widely supported by several studies on the SNARC effect: small numbers elicit faster responses when presented on the left, while responses for larger numbers are faster when these are presented on the right. The SNARC effect clearly demonstrates how numbers are mapped along a spatial representation going from left to right^11–16^.

Another class of evidence reporting interactions amongst space, time and number comes from studies on adaptation to motion – that is, changes of position over time. For example, it has been reported that motion adaptation robustly affects localization of the adapted stimuli: viewing a drifting grating or rotating windmill for several seconds causes a subsequent grating to appear displaced from its veridical position^17–19^. Motion-induced distortions of perceived position are independent from motion aftereffects (MAEs): even when the MAE has already vanished or it is annulled by moving the adapted stimulus in the opposite direction as the adapter, displacements of the perceived position still occur^18^. More recently, it has been demonstrated that adaptation to visual motion also distorts the perceived duration of visual stimuli. A prolonged exposure to a fast translating grating yields a robust compression of the perceived duration of a stimulus subsequently presented in the adapted location^20–23^. Compression of perceived duration is robust (on average around 30–40%), spatially localized and likely to occur at multiple levels of the visual hierarchy^24^, as evidence supporting early (sub-cortical)^20,25,26^ as well as late processing stage^21,27,28^ has been reported. Moreover, compression of perceived duration is specific for adaptation to translational motion, as adaptation to motion profiles entailing more than a motion direction at the same time (i.e. circular or radial motion) leaves duration estimates veridical^29^.

While the effects of motion adaptation on spatial and temporal processing are clear, less is known about the impact of a sustained exposure to visual motion on perceived numerosity. According to the ATOM theory, the processing of magnitude dimensions such as space, time and numerosity would exploit partially overlapping neural substrates. If so, changes in the activity of these shared mechanisms induced by motion adaptation (tapping on the processing of both space and time) should provide distortions also in the processing of the seemingly unrelated dimension of numerosity. Recently, Schwiedrzik and colleagues^30^ tackled this issue by investigating whether motion adaptation distorts the perception of non-symbolic numerosity. They found that while adaptation to leftward motion yields overestimation for small numbers, rightward adaptation leads to an underestimation of large numbers. Moreover, by means of a series of control experiments, the authors concluded that the best candidate as a neural substrate for these phenomena is the homolog of monkey’s parietal area LIP, an area that has been previously been reported to be strongly involved in magnitude processing^1,2,31–35^.

Schwiedrzik *et al*.’s study provides a clear demonstration of the interactions between spatial and numerical magnitudes. However, it is focussed on perceived numerosity being distorted by adaptation to a *given motion direction*. In other words, the study assessesed whether the typical repulsive aftereffect induced by motion direction adaptation generalises to symbolic numerosity perception. However, direction aftereffects (DAEs) are quite common phenomena that have been reported even across senses (i.e. adaptation to horizontal auditory motion induces significant visual motion aftereffects^36^) to make it uncertain whether such effects are perceptual in nature (for example due to sensory integration) or induced by some sort of response/decisional bias. A different approach would be to investigate whether motion adaptation *per se* – that is, a sustained exposure to a given number of events (defined as changes of position over time) – affects approximate numerical estimates independently of DAEs. Here, we devised a paradigm similar to that previously exploited to investigate the effects of motion adaptation on perceived duration^20,21,23,25,37^. Doing so, we aimed to draw a parallel between the effects of motion adaption on perceived numerosity and those reported on perceived duration.

We measured subjects’ ability to perform a numerosity discrimination task and assessed whether, and to what extent, subjects’ performance was affected by adaptation to visual motion. We investigated motion adaptation to both fast (20 Hz) and slow (5 Hz) speed for two different motion profiles – translational and circular motion – while counterbalancing motion directions to avoid net directional aftereffects. Perceptual estimates of stimulus duration are strongly affected by adaptation to fast translational motion; however, they remain rather veridical for adaptation to slow drifting and to complex motion profiles (i.e. circular or radial motion) regardless of the adapting speed^20,21,25,27,29^. Testing for multiple motion profiles is also a tool to get hints about which neural substrate underpin these adaptation aftereffects. If adaptation operates at a late stage at the level of a generalized magnitude system – allegedly to be located in parietal cortices such as IPS – motion adaptation might affect numerosity estimates similarly regardless of the motion profile taken into account. Conversely, if motion adaptation acts on perceived numerosity *before* the magnitude system – at the level of dimension-specific processing stages upstream to the parietal cortex – we might expect different outcomes for adaptation to different motion profiles, reflecting the involvement of different motion mechanisms selective for translational or circular motion – similarly to what has been previously observed on time perception^29^.

## Results

**Figure 1:**
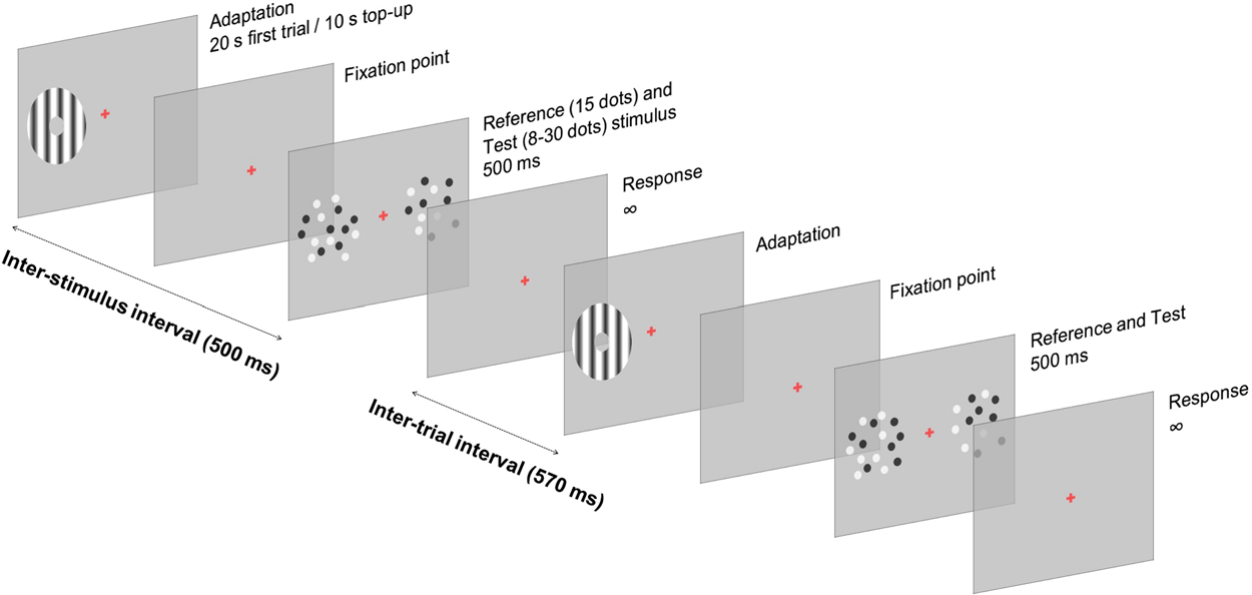
Experimental procedure. Example of the adaptation procedure in the translational motion condition. Each trial started with the presentation of the adaptor stimulus. After the adaptation period, two dot-array stimuli were presented on the screen: a reference (fixed numerosity), presented in the adapted location, and a test (variable numerosity) stimulus presented in a neutral un-adapted location. Participants were asked to discriminate which one of the two dot arrays contained the larger number of dots. After providing a response, the next trial started automatically after a brief blank interval. A similar procedure (except for the kind of adapter stimulus) was applied to investigate the effect of adaptation to circular motion. Stimuli are not reported in scale.

Figure 1 shows an example of the procedure employed in our adaptation conditions. In each trial, participants were first adapted to either fast (20 Hz) or slow (5 Hz) translational or circular motion (each condition tested in separate sessions), and then they were asked to discriminate which one of two simultaneously presented stimuli (one on the left and one of the right of a central fixation point) contained more dots (for details see *General Procedure* in *Methods*).

**Figure 2:**
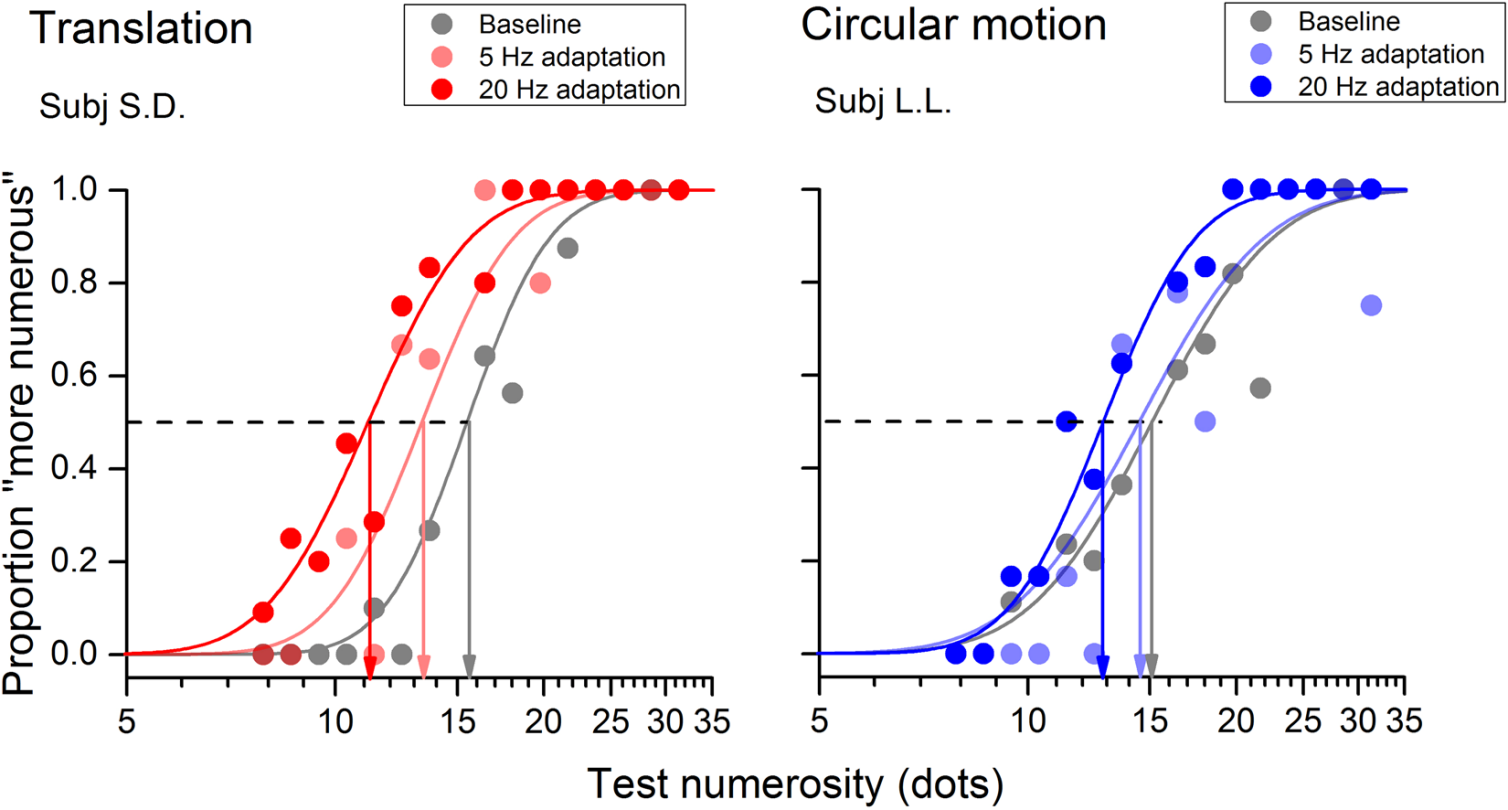
Psychometric functions for the numerosity discrimination task. Psychometric functions showing the proportion of trials where the test stimulus appeared more numerous than the reference stimulus as a function of the numerosity of the test. In each trial the numerosity of the test stimulus was chosen by means of an adaptive staircase procedure (see *General procedure* in *Methods*), whilst the reference stimulus, presented in the adapted location, had always a fixed numerosity of 15 dots. The panel on the left refers to adaptation to translational motion and the panel on the right to adaptation to circular motion. Data in grey indicate the baseline condition in which the discrimination task was performed without adaptation. Data in light colour refer to conditions in which the adapting speed was slow (5 Hz) and full coloured lines refer to adaptation to fast motion (20 Hz). Despite the adapting motion profile being translational or circular, motion adaptation induced a compression of perceived numerosity as indicated by the leftward shift of the psychometric functions. However, adaptation to translational motion induced a much more robust compression of perceived numerosity to suggest a complex – motion profile-dependent – interaction between motion and numerosity processing.

Figure 2 shows psychometric functions for two subjects obtained by plotting the proportion of trials in which the test stimulus (unadapted) was judged as more numerous than the reference (adapted; Fig. 1), as a function of test numerosity. The left and the right panels of the figure show the results for the condition concerning adaptation to translational and circular motion, respectively. Adaptation to translational motion strongly compressed perceived numerosity as shown by the consistent leftward shift of the red curves in the left panel of Fig. 2. However, the aftereffects co-varied with the adapting speed as shown by adaptation to slow translation compressing perceived numerosity to a much lesser extent.

**Figure 3:**
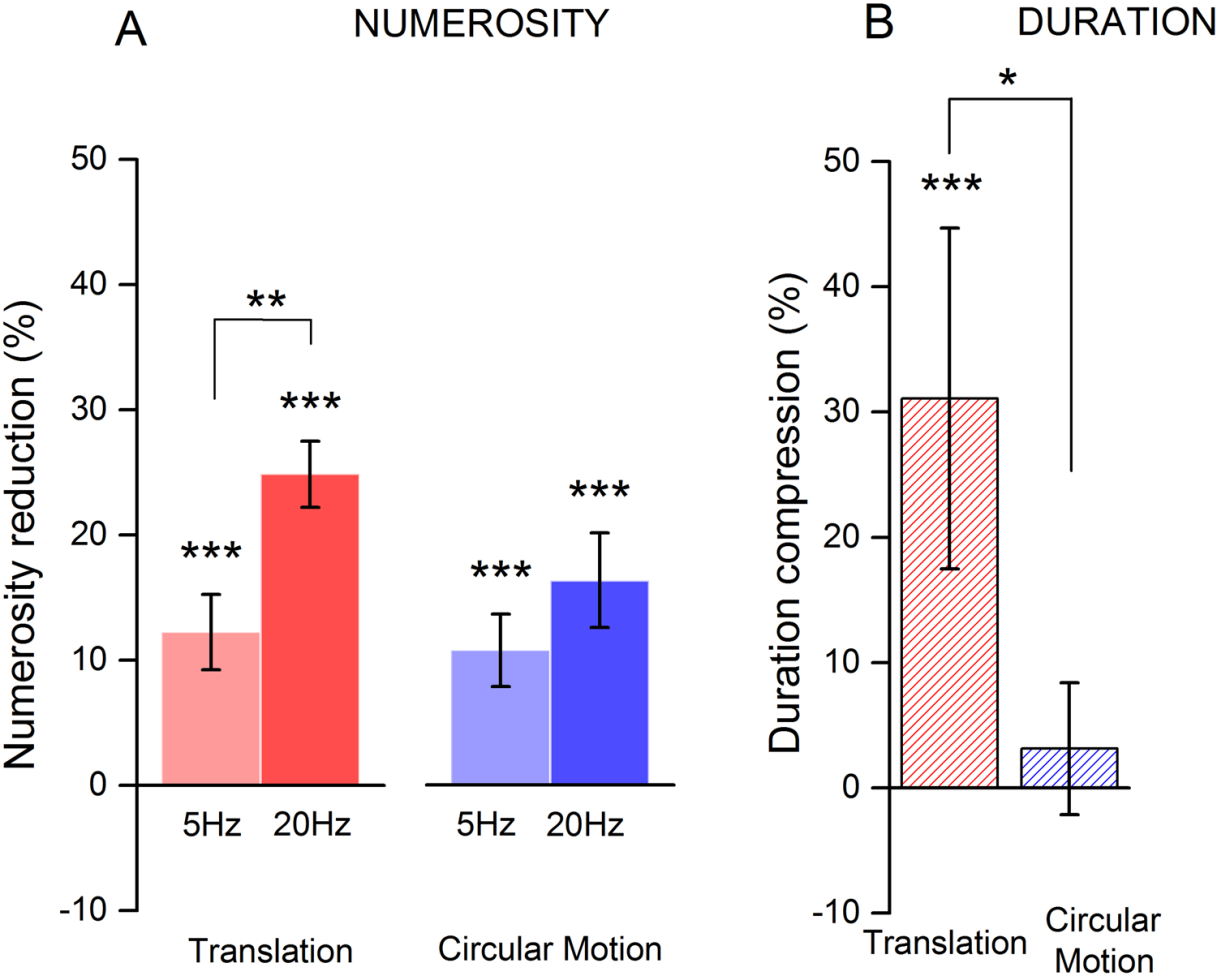
Average numerosity compression for adaptation to translational or circular motion. Panel A: Reduction in perceived numerosity induced by adaptation to translational (in red) or circular motion (in blue). In all conditions, numerosity reduction was calculated as the difference between post-adaptation and baseline PSEs, normalized by the baseline. Adaptation to fast translational motion strongly compressed perceived numerosity by on average 20–25%. Fast rotational motion yielded a robust compression (around 15–17%) but it was smaller than that induced by fast translation. Perceived numerosity was also distorted by adaptation to slow circular and translational motion, with the two motion profiles providing similar compression (around 10%). Panel B: Reduction of stimulus perceived duration induced by adaptation to fast translational (red hatched bar) or circular (blue hatched bar) motion (data from Fornaciai *et al*.^29^). Whilst adaptation to translation robustly affected perceived duration and numerosity (in both cases providing a compression of about 20–30%), adaptation to circular motion significantly distorted the estimates of perceived numerosity without affecting perceived duration. Asterisks above each bar refer to tests comparing pre- and post-adaptation PSEs. Asterisks between bars refer to tests comparing the magnitude of adaptation across different conditions. Error bars represents S.E.M. *p < 0.05, **p < 0.01, ***p < 0.001.

Adaptation to fast circular motion also yielded a significant compression of perceived numerosity even if numerosity distortions were weaker than those triggered by fast translational motion. On the contrary, slow circular motion slightly affected perceived numerosity as shown by the light blue curve in the right panel of Fig. 2 rather overlapping the baseline’s grey curve. To better quantify adaptation effects, we measured the amount of compression in perceived numerosity induced by motion adaptation as a normalized difference between baseline and post-adaptation PSEs (see *Methods*). Figure 3A shows the amount of compression averaged across all subjects for adaptation to both fast and slow translational and circular motion.

Fast translational motion compressed perceived numerosity by about 25%, whereas slow translation provided around one half of such an effect, with compression being about 12%. Adaptation to circular motion also affected perceived numerosity but to a lesser extent. A sustained exposure to fast circular motion reduced perceived numerosity by about 17%, while slow circular motion (5 Hz) provided a compression similar to that achieved for slow translational motion (around 12%). A series of paired-sample t-test showed that post-adaptation PSEs were significantly lower compared to baseline measures, in all the adaptation conditions taken into account: translation 5 Hz (t_(13)_ = 3.97, p < 0.001), translation 20 Hz (t_(13)_ = 8.92, p < 0.001), circular motion 5 Hz (t_(13)_ = 3.92, p < 0.001); circular motion 20 Hz (t_(13)_ = 4.23, p < 0.001). Furthermore, the magnitude of the effects across different conditions was compared using a two-way ANOVA by using as main factors “speed” and “motion profile”. The results showed a significant main effect of speed (F_(1,55)_ = 9.25, p = 0.004) whilst neither the effect of motion profile (F_(1,55)_ = 2.77, p = 0.102) nor the interaction amongst the two (F_(1,55)_ = 1.38, p = 0.245) was found to be significant. However, a post-hoc multiple comparison test (Holm-Sidak multiple comparison) showed that while adaptation to translational motion at different speeds provides significantly different effects (i.e. larger effect with 20 Hz adaptation; t_(13)_ = 3.04, p = 0.004), such a difference was not significant for circular motion (t_(13)_ = 1.32, p = 0.193).

The magnitude of changes in perceived numerosity induced by motion adaptation varied consistently according to the adapting motion profile as well as the adapting speed. In general, fast motion provided changes in perceived numerosity more robust than slow motion and the same held for adaptation to translational than circular motion. Were these adaptation-induced changes in accuracy (PSEs) mirrored by changes in judgements precision (JNDs)? As it should be expected in terms of scalar variability (higher precision for lower numerosity) the most consistent reduction in JNDs were found for the conditions providing the strongest numerosity reduction, namely, those regarding adaptation to fast motion. The difference in JND was highly significant after adaptation to fast translational motion (baseline vs 20 Hz translation: p < 0.001) and marginally significant after adaptation to fast circular motion (baseline vs 20 Hz circular: p = 0.06). On the contrary, adaptation to slow motion did not provide significant changes in precision neither for translation (baseline vs 5 Hz translation: p = 0.24) nor for circular motion (baseline vs 5 Hz circular: p = 0.409). Moreover, there was no significant difference between post adaptation JNDs induced by fast motion (translation 20 Hz vs. circular 20 Hz: t(13) = −1.44, p = 0.172) or slow motion (translation 5 Hz vs. circular 5 Hz: t(13) = −1.42, p = 0.177).

**Figure 4:**
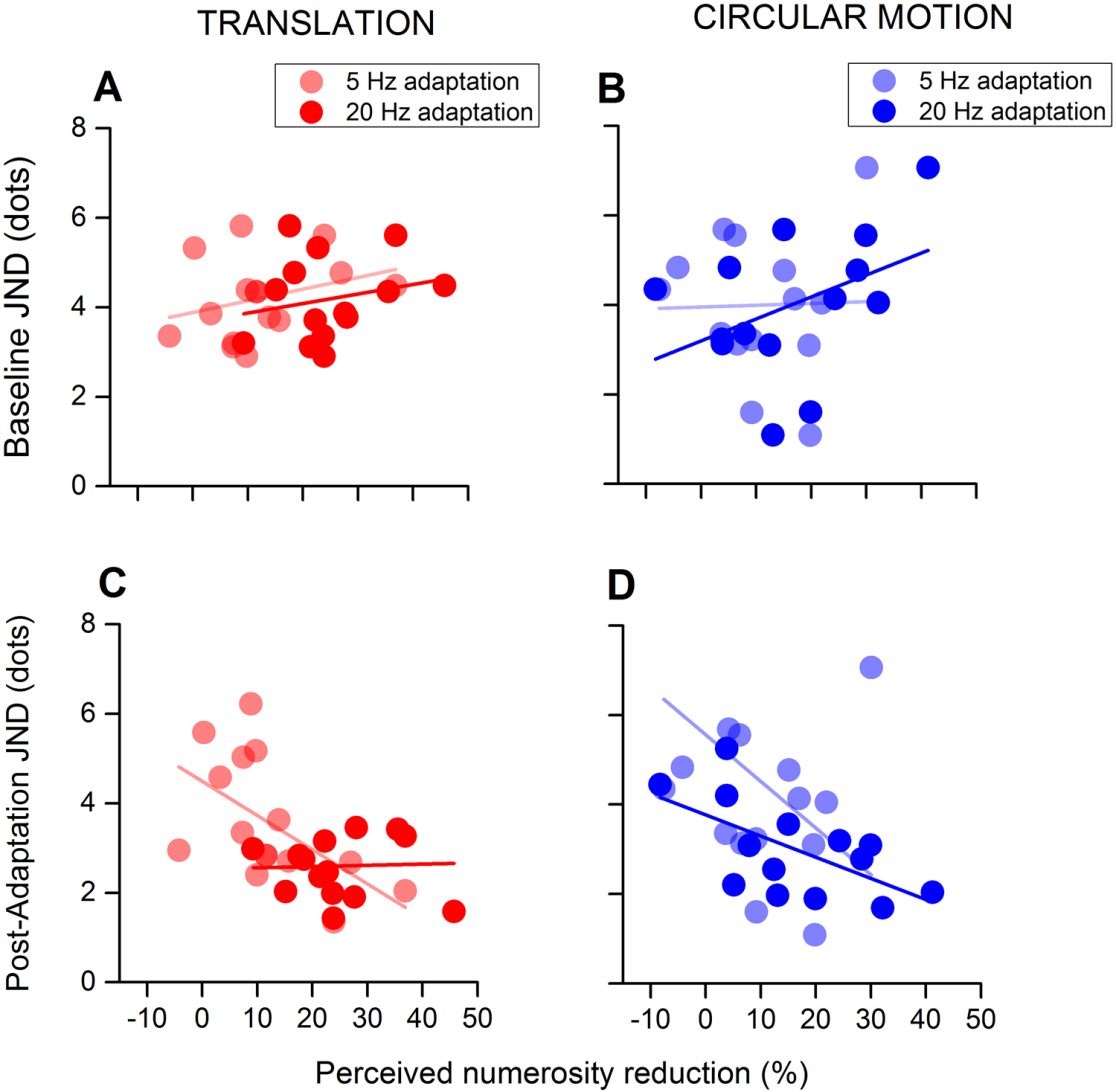
The relation between baseline and post-adaptation JNDs and adaptation effects. (**A**,**B**) Pre-adaptation (baseline) sensitivity for numerosity discrimination (JND) plotted as a function of post adaptation numerosity reduction for (**A**) translational and (**B**) circular motion. None of the correlations concerning baseline JND turned out being statistically significant, suggesting independency between precision in numerosity discrimination and susceptibility to motion adaptation. (**C,D**) Correlations between post-adaptation JND and adaptation magnitude for translational and circular motion (**C** and **D** respectively). In most cases post-adaptation JNDs negatively correlated to the magnitude of the adaptation to indicate a concurred reduction of PSEs and JNDs. This in turn reflect an increase in precision for lower perceived magnitude in line with Weber’s law.

However, adaptation aftereffects also varied widely across participants. Might this variability be related to inter-individual difference in sensitivity to numerosity? In a recent study, Van der Burg and colleagues^38^ reported that the size of audio-visual recalibration, induced by exposure to audio-visual pairs, strongly correlated with the width of the simultaneity window. That is, the more tolerant the simultaneity judgments, the stronger the recalibration effect^38^. To investigate the relationship between sensitivity in numerosity discrimination and adaptation magnitude, we compared the precision of baseline numerical judgments (JNDs) with the magnitude of the adaptation effect. The results are shown in Fig. 4A,B for both adapting profiles and speeds.

For adaptation to translational motion (both 20 and 5 Hz) and slow circular motion, correlations were found to be small and far from being statistically significant (translation 5 Hz: r = 0.30, p = 0.27; translation 20 Hz: r = 0.22, p = 0.42; circular motion 5 Hz: r = 0.02, p = 0.92). A more consistent correlation emerged for adaptation to fast circular motion (r = 0.42) even though statistical significance was not reached (p = 0.13). We also analysed correlations between the reduction in perceived numerosity and *post*-adaptation JNDs (Fig. 4C,D). Post-adaptation JNDs significantly decreased as compression of perceived numerosity increased for circular motion (5 and 20 Hz) as well as slow translation. In all three conditions, the bigger the numerosity underestimation, the higher the precision (Pearson correlations, translation 5 Hz: r = −0.56, p = 0.029; circular motion 5 Hz: r = −0.63, p < 0.015; circular motion 20 Hz: r = −0.60, p = 0.02). The results of a significant negative correlation between post-adaptation JNDs and adaptation magnitude, indicate that Weber-Fechner’s law is obeyed: the precision of perceptual estimates is proportional to the magnitude of the sensory input. Thus, the effects of motion adaptation on perceived numerosity is likely to be perceptual in nature and not due to non-perceptual factors (i.e. changes in decision criteria). However, post adaptation JNDs did not correlate with adaptation magnitude for fast translational motion (Pearson correlations, translation 20 Hz: r = −0.07, p = 0.82). A possible explanation for this lack of correlation is that it may be due to a lack of variability in the data. Indeed, JNDs after adaptation to fast translation – that is, the condition triggering the strongest compression of perceived numerosity – resulted to be generally very small, regardless the magnitude of the adaptation effect (see Fig. 4C). This might suggest the existence of maximum level of precision that cannot be exceeded with this edge effect that might prevent correlations to occur.

**Figure 5:**
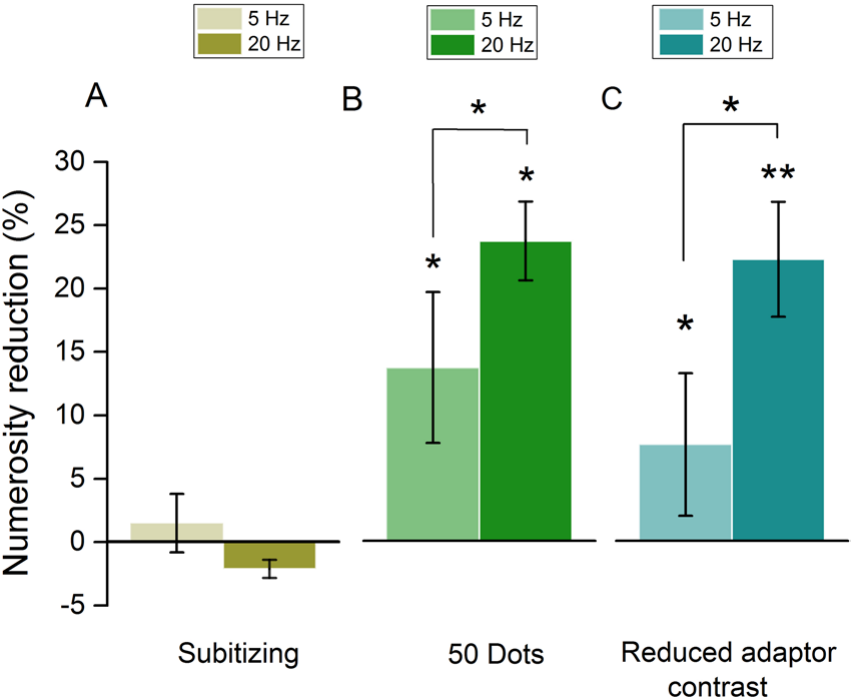
Adaptation to different numerosity regimes and to low contrast adapters. Panels A,B: Perceived numerosity reduction induced by motion adaptation in the subitizing range (reference numerosity = 3 dots; panel A) or the estimation range (numerosity 3 times higher than in Exp 1; panel B). Motion adaptation did not affect perceived numerosity in the subitizing range. On the contrary, perceived numerosity in the estimation range was found to be robust regardless reference numerosity being 15 (as in Exp 1) or 50 dots. (C) Motion adaptation-induced numerosity reduction effects after adaptation to translational motion, in the condition where the adaptor contrast was reduced to 50%. Error bars represent S.E.M. *p < 0.05, **p < 0.01.

A possible concern about the aforesaid results is that motion adaptation might affect perceived numerosity indirectly; for example, via a non-specific masking mechanism. This idea generates several hypotheses that we tested with a series of control experiments. We first addressed the issue of whether adaptation is specific for a given numerosity range. Recent results suggest that numerosity perception is mediated by different mechanisms: an errorless mechanism for numerosity within the subitizing range (1–5); a mechanism obeying Weber’s law for higher numerosities in which the number of items can only be approximately estimated; and a texture-density mechanism following a square root law operating when items are too dense to be individually segregated^39,40^. Here we investigated the effects of adaptation to translational motion on numerosity perception for a reference numerosity about three times higher than that exploited in Experiment 1 (from n = 15 to n = 50) but still falling in the estimation range as confirmed by a lack of significant difference in the weber fractions for numerosity discrimination with a reference of 15 and 50 dots (one-tailed, paired sample t-test on the 7 subjects that participated to the two experiments; t_(6)_ = 0.7874, p = 0.78). Furthermore, we also investigated the effect of adaptation to fast translational motion for numerosity falling within the subitizing range (n = 3). Predictions are clear: if motion genuinely affects perceived numerosity, the effect on the higher numerosity range should be comparable to those observed in Experiment 1 whilst estimates in the subitizing range should not be affected by adaptation and remain veridical. Indeed, previous studies showed that subitizing mechanisms are virtually immune to adaptation, unless attentional resources are diverted, for example by presenting a concurrent additional task^41,42^. Panels A and B of Fig. 5, show adaptation effects for a reference numerosity of 3 and 50 dots respectively. Motion adaptation strongly affected discrimination for numerosity around 50 dots, with the reduction in perceived numerosity being similar to that achieved for a reference numerosity of 15 dots (see Fig. 3). Namely, adaptation to translational motion yielded a reduction of about 25% for translation at 20 Hz and of about 14% for slow (5 Hz) translation. Both effects resulted to be statistically significant as shown by one-way R.M. ANOVA with Holm-Sidak multiple comparison procedure versus control: baseline vs. 20 Hz adaptation, t_(5)_ = 5.867, p < 0.001 and baseline vs. 5 Hz adaptation, t_(5)_ = 3.432, p = 0.006), to indicate that both adaptation conditions yielded a significant shift of the PSEs compared to baseline. On the contrary, motion adaptation did not provide any significant change in perceived numerosity when the numerosity range was kept in the subitizing range (F_(2,4)_ = 2.330, p = 0.159) as shown by panel A of Fig. 5.

Finally, we tested whether adaptation compresses perceived numerosity via a spurious effect, that is, by reducing the visibility of the adapted stimuli. Despite this hypothesis had already been partially falsified by the results on the subitizing range (a change in dots visibility should be independent from the numerosity regime), we devised a new version of Experiment 1 to investigate the effect of adaptation to fast translational motion (the most effective condition), with adaptors at a reduced contrast: from 90% to 50%. The idea is to leverage on the finding that when adaptors are lower in contrast than test stimuli, perceived contrast (and thus visibility) of the latter remains unaffected^43^. Panel C of Fig. 5 shows the results for adaptation to low contrast adaptors. In general, the pattern of results for low and high contrast adaptors is quite similar. Even with adaptors with a Michelson contrast of 50%, fast translational motion (20 Hz) distorted perceived numerosity by about 20%, while slow translation (5 Hz) yielded a weaker distortion, about 7–8%. A one-way R.M. ANOVA confirmed that in both conditions post-adaptation PSEs were significantly different from baseline (Holm-Sidak multiple comparison versus control [“baseline”]: baseline versus 5 Hz: t_(4)_ = 2.526, p = 0.035; baseline versus 20 Hz: t_(4)_ = 5.057, p = 0.002), and that effects provided by adaptation to fast translational motion were stronger than those observed for slow translation: paired sample t-test on numerosity reduction values, t_(4)_ = 2.943, p = 0.0211. Taken together, the results concerning the magnitude of adaptation aftereffect in the subitizing range (Fig. 5, panel A), and those for low contrast adapters (Fig. 5, panel C), congruently support the idea that the effects of motion adaptation on perceived numerosity are unlikely to be accounted for in terms of visibility reduction of the adapted stimuli.

## Discussion

In the present study, we investigated the effects of visual motion adaptation on perceived numerosity. Our results demonstrate that a sustained exposure to either translational or circular motion compresses numerosity estimates of stimuli subsequently presented in the adapted location. The magnitude of adaptation is temporal frequency-dependent with adaptation to fast motion (20 Hz) providing a much stronger effect than adaptation to slower motion (5 Hz). This adaptation trend appears to be dependent on the adapting motion profile with fast translation being more effective than fast circular motion, even if this difference was not found to be statistically significant.

It might be argued that motion adaptation does not affect stimulus numerosity directly but by changing the number of perceived dots via spurious effects on non-numerical feature such as their texture-density. However, several arguments speak against this hypothesis. First, Cicchini and colleagues^44^ have recently demonstrated that in many conditions in which visual stimuli are clearly segregated from each other (as they were in the experiments reported here), even in case the subjects are not directly instructed to make numerosity judgments (but they were here), they show a tendency to spontaneously judge stimulus numerosity rather than area or density. In other words, it turned out that under many conditions, numerosity is the dimension the observers are more sensitive to and thus the one they mostly rely on for their judgments^44–47^. On top of this, Anobile and colleagues^39^ have provided evidence about perception of numerosity and density being two completely distinct processes. The numerosity process takes place when items are clearly distinguishable from each other (as they were in the present study), density when they are not (i.e. too cluttered to be segregated from each other). Anobile and colleagues also provided a quantitative “index” to discriminate between the regime of numerosity and texture-density perception and it is related to the Weber Fraction (WF) values. Numerosity perception obeys Weber’s law with discrimination thresholds varying linearly with the number of items to estimate. On the contrary, texture-density follows a square-root law with the Weber fractions getting smaller as the number of objects increases (see also Figure 6 in this review^48^). What was the perceptual regime involved by our discrimination tasks? We measured WFs for our discrimination tasks with a reference numerosity of 15 and 50 dots and we found WFs in the two conditions to be rather identical to suggest that in both these tasks subjects judged stimulus numerosity not texture-density. To summarise, the extant literature supports the idea that in experimental conditions similar to those exploited in the present study, subjects mostly base their judgments on stimulus numerosity rather than density or area. In our opinion this makes more parsimonious to think of motion adaptation as affecting the perceptual process likely to be involved in the task, namely, numerosity instead of the perceived texture-density of the adapted stimuli.

However, another possibility for an indirect, spurious effect of motion adaptation on perceived numerosity is that adaptation may reduce the perceived contrast (visibility) of the adapted stimuli, and this in turn would provide an underestimation of stimulus numerosity. None of our results support this idea. First, motion adaptation does not affect numerosity perception within the subitizing regime (<5 items). However, a low-level effect such as a reduction in perceived contrast should affect the stimulus’ visibility regardless of its numerosity, making it possible for the observer to miss one or more items even at extremely low numerosities. Second, adaptation to low-contrast adapters provides rather identical effects than high-contrast adapters. However, it has been reported that perceived contrast of high contrast stimuli is minimally affected by adaptation to low contrast adaptors^43^, suggesting that changes in perceived numerosity induced by motion adaptation are genuine, not spurious consequences of changes of stimuli visibility.

Even if the effect of motion adaptation on numerosity is genuine, it may not be perceptual in nature but instead related to some unspecific cognitive or decisional factor. Two lines of evidence stand against this hypothesis. First, the effect is spatially localized. We adapted a region of space on the left of a central fixation point. Then, we *simultaneously* presented a reference and a test stimulus in the adapted and neutral location, respectively. The changes in PSEs found in the adaptation condition, relative to the baseline, suggest that adaptation selectively affects one of the two stimuli: if motion adaptation affected both, we would not have recorded any shift of the PSEs. Second, in most conditions we found a consistent negative correlation between numerosity compression and post-adaptation JNDs. In other words, when the subjects perceived fewer stimuli as a consequence of motion adaptation, they also became more precise in line with the scalar variability of Weber’s law.

At which stage of visual processing might motion and numerosity perception interact? A first hypothesis is that motion and numerosity interact at an early stage of visual processing. Fornaciai and colleagues^46^ have recently found a signature of numerosity processing at around 100 ms after stimulus onset, with this activity likely arising from early visual areas such as V2 or V3. Intriguingly, these two areas have been recently reported to be highly sensitive to global motion^49^, and this in turn makes V2 and/or V3 perfect candidates to underpin interactions between motion and numerosity processing. Finally, the idea that motion mechanisms might affect numerosity processing at early stage is in line with evidence that numerosity estimates are strongly affected by the spatiotemporal properties of visual stimuli^50^. Perceived numerosity of clouds of moving dots randomly changing their locations, either abruptly or smoothly, was found to be systematically overestimated, but with smooth motion yielding a significantly reduced effect. Taken together, these results indicate a role of motion integration in numerosity processing and open up to the possibility that compression of perceived numerosity induced by motion adaptation might be the consequence of a normalization process yielded by the motion mechanisms. Indeed, Fornaciai and Park^50^ interpreted the role of motion-sensitive areas as a normalization process preventing the overestimation of moving objects. In these terms, numerosity underestimation might be explained as an exaggerated normalization response. In other words, after prolonged exposure to fast motion, subsequent stimuli might be still affected by the normalization process, that in the case of static objects would result in a net underestimation of the actual numerosity.

An alternative hypothesis is that motion and numerosity interact at a relatively late stage, as proposed by theoretical frameworks suggesting the existence of neural machinery dedicated to magnitude processing (i.e. a theory of magnitude – ATOM^2^) in the parietal lobe^31–34^. When the activity of these neural circuits mediating a common representation of quantity is affected, processing of multiple perceptual dimensions may be distorted. Indeed, one of the hypotheses tested in this study is that perceived numerosity might be affected by motion adaptation similarly to perceived duration^29^. Our results reveal that adaptation to fast translational motion compresses perceived numerosity of about the same extent (20–25%, see Fig. 3) as it does with duration. This result is consistent with the idea of a “common magnitude system” processing spatial, temporal and numerical information as initially proposed by Walsh^2^. These results also complement those by Schwiedrzik and colleagues^30^ that showed motion direction to strongly bias the perceived number of items, as if the adapting direction would provide a shift along the mental number line. For example, adaptation to leftward motion caused overestimation whilst rightward adaptation yields underestimation. These results provide evidence for an intimate link between the perception of space and number. However, the similarities between the effects of motion adaptation on perceived duration and numerosity are limited to fast translational motion and do not generalize to the other conditions tested in the present study. For instance, it has been reported that adaptation to circular motion does not affect duration estimates, leaving them veridical^29^. Conversely, we found that adaptation to circular motion robustly affected perceived numerosity (up to 16% for fast circular motion). Moreover, slow translational and circular motion (5 Hz) – both known to have little or no effect on perceived duration^20,22,29^ – were instead found to significantly compress perceived numerosity even if to a lesser extent than fast motion (around 10% vs 20–25%).

Even if a more solid argument in favour of a common magnitude system for the perception of motion and numerosity would require more evidence, it is clear that interactions amongst these dimensions occur and they also transcend the perceptual domain. Our group has recently demonstrated that adapting to self-generated actions does consistently affect the representations of numerosity of external events^51^. A short period of rapid finger-tapping, without sensory feedback, biased the subjects to underestimate the number of presented visual stimuli whilst a period of slow tapping yielded an opposite effect. These aftereffects were found to be perceptual in nature as shown by their spatial selectivity: numerosity estimates were distorted near the tapping region but remain veridical elsewhere. Moreover, the reference frame of these effects was in external, not hand centred coordinates, as shown by conditions in which subjects trapped with their hand being crossed along the body midline. The results from Anobile and collaborators^51^, and those of the present study, open up to an interesting hyphothesis. Self-generated motion of body limbs, or motion of the stimuli in the external space, might automatically trigger the processing of quantity information such as the number of repetitions in a tapping routine or the number of position changes of white bars in a drifting gratings. This quantity information would activate numerosity mechanisms yielding the adaptation aftereffects reported by our group in the present and previous studies. A prediction of such hyphotesis would be that adaptation to several repetitions of self-produced movements may distort stimuli perceived duration as it has been been reported to occur for adaptation to fast visual motion. Further studies will be needed to test such prediction.

## Conclusions

Overall, our results suggest a close link between motion processing and numerosity perception, in line with previous studies^30,50^. This relationship between motion and numerosity supports the idea of similar, yet partially dissociable, pathways for the processing of different magnitudes. However, the difference between the effect of complex motion adaptation on perceived numerosity and perceived duration^29^ suggests that magnitude-specific effects of motion adaptation occur at different levels of the visual processing stream.

## Methods

### Participants

A total of 24 subjects (13 females; age ranging from 21 to 30 years) participated in either one or multiple conditions of the study. All the participants were naive to the purpose of the study, with the exception of authors I.T. and M.F., who participated to all experimental conditions. Experimental procedures were approved by the local ethics committee (Comitato Etico Pediatrico Regionale—Azienda Ospedaliero-Universitaria Meyer—Firenze, FI), and were in line with the declaration of Helsinki. All participants gave their written informed consent.

### Stimuli

All visual stimuli were generated with the Psychophysics Toolbox V.3^52^ for MatLab (version 2010b) running on a PC computer, and presented on a Barco CRT monitor (Barco Calibrator Line), subtending 40 × 30 degrees of visual angle at the viewing distance of about 57 cm. Screen resolution was set to 800 × 600 pixels with a refresh rate of 100 Hz. In the numerosity discrimination task stimuli consisted of clouds of dots arranged within an invisible annulus (the inner and external edge located at 1° and 5° from the stimulus center, respectively) with a minimum inter-dot distance of 0.75 deg. Adapters consisted of either translating or rotating patches moving at high (20 Hz) or low (5 Hz) speed, with each combination of adapting motion profile and temporal modulation tested in separate sessions. Translating adapters were luminance modulated gratings (spatial frequency = 1 cycle per degree) drifting horizontally. Circular motion adapters were windmill-like rotating gratings, with spatial frequency increasing from 0.5 to 1.2 cpd from the outer to the inner border respectively. Both classes of adapters were windowed within an annular mask (distance from the center to the inner and outer edge equal to 1 deg and 5.5 deg respectively), with borders blurred by a Gaussian smoothing (spatial constant equal to 0.15 deg) and were presented either with a Michelson contrast of 90% or 50% (tested in separate sessions). To prevent perceived numerosity to be affected by motion direction aftereffects, all adapters reversed the moving direction every 2 seconds. Given that they were presented for 20 s on the very first trial with a top up of 10 s in all following trials this implied a total of 10 and 5 reversals respectively. The annular shape was chosen to keep stimuli consistent with previous studies on time perception^29^.

### General procedure

On each trial, subjects were simultaneously presented with two stimuli, a test and a reference, both presented at a horizontal eccentricity of 10° respectively to the right and to the left of a central fixation point. The subjects’ task was to indicate which stimulus contained more dots by pressing the appropriate key on a keyboard (2AFC – 2-Alternatives Forced Choice). The numerosity of the reference stimulus was kept constant (15 dots) whilst the test numerosity varied from trial to trial according to an adaptive QUEST staircase^53^ within a range of ±0.3 log units relative to the reference numerosity. Participants typically completed at least two blocks of 50 (baseline) or 30 (adaptation) trials, which were usually sufficient to achieve robust estimates of perceptual performance. Occasionally (i.e. in case of outlier responses during the main staircase procedure), we asked participants to complete 1–2 more blocks to reduce noise in accuracy estimates. We measured subjects’ accuracy by means of the point of subjective equality (PSE) defined as the median of the best-fitting cumulative Gaussian function to the data representing the percentage of responses “test as more numerous” against test physical numerosity. Precision was instead measured as the just noticeable difference (JND), defined as the standard deviation of the underlying Gaussian function. On separate sessions, subjects performed numerosity discrimination after being adapted to fast (20 Hz) or slow (5 Hz) translational or circular motion.

In the adaptation sessions, each trial started with the presentation of the adaptor stimulus, displayed in the left portion of the screen with a horizontal eccentricity of 10 deg (same location as the following reference stimulus). After 500 ms from the adaptor offset, test and reference were presented according to the procedure described above. To assess whether and to what extent motion adaptation affects perceived numerosity, we calculated a perceived numerosity reduction index, defined as the difference between post-adaptation and baseline PSE, normalized by this latter and transformed in percentage:

1 $${\rm{Perceived}}\,{\rm{numerosity}}\,{\rm{reduction}}=-\,1\ast (({\mathrm{PSE}}_{{adapt}}-{\mathrm{PSE}}_{{baseline}})/{\mathrm{PSE}}_{{baseline}})\ast \mathrm{100}$$

with PSE_*baseline*_ and PSE_*adapt*_ representing participants’ accuracy in the baseline and adaptation condition, respectively. Note that as compressive effects result in negative effects when measured as the difference between baseline and post-adaptation PSEs, we reversed the sign of the effect when calculating the magnitude of perceived numerosity reduction. Fourteen participants were tested in the main experiment.

A series of control experiments, only concerning adaptation to translational motion, were carried out to test whether motion adaptation aftereffects are numerosity range-dependent and whether they might be accounted for in terms of any kind of masking effects. To these aims we tested, in separate conditions, the effect of motion adaptation on perceived numerosity on either a higher range (around 50 dots) or a lower range (3 dots to fall within the subitizing regime). Finally, in additional conditions we reduced the adaptor contrast from 90 to 50%, in order to minimize the effect of contrast masking on perceived numerosity. Six additional participants were tested in these control experiments concerning discrimination of larger numerosities, low numerosity (within the subitizing range), and adaptation to low contrast stimuli. For each of these experimental conditions we generally collected from 3 to 5 sessions of data each of which containing 30 trials.

### Data availability

The datasets generated during the current study are available from the corresponding author on reasonable request.
